# An energy-centric perspective on safety for physical human-robot interaction under ISO guidelines

**DOI:** 10.3389/frobt.2026.1901111

**Published:** 2026-10-26

**Authors:** Riccardo Zanella, Federico Califano, Stefano Stramigioli

**Affiliations:** Robotics and Mechatronics, University of Twente, Enschede, Netherlands

**Keywords:** human robot collaboration (HRC), human robot interaction (HRI), ISO/TS 15066, safe robotics, safety

## Abstract

The International Organization for Standardization (ISO) defines safety as the ability of a robotic system to operate within limits that reduce risks to humans to an acceptable level. However, ISO standards (such as ISO/TS 15066 and ISO 10218–2) contain ambiguities that may lead to inconsistent risk assessments or hazard underestimation. This Perspective Article presents an energy-centric viewpoint on safety in physical human-robot interaction (pHRI). Building on our previous critical review of pHRI safety constraints, the article isolates and explicates one central theme emerging from that analysis: the role of mechanical energy in interpreting ISO/TS 15066 transient-contact limits. Specifically, we discuss how pain-related force limits can be mapped to admissible elastic energy stored in tissue deformation, why the instant of maximum deformation is central for transient-contact assessment, how this interpretation changes under free-recoil and potential-clamp conditions, and why energy-based metrics remain insufficient as standalone descriptors of pain perception or sustained-contact risk. We conclude by outlining open challenges and future research directions for leveraging energy-centered metrics toward safe and effective human-robot interaction.

## 1 Introduction

Over the past 2 decades, robotics has shifted from traditional industrial setups relying on physical separation to a broader paradigm of human-robot coexistence. A particular challenge arises when the mutual exchange of information between human and robot involves physical contact, giving rise to the topic of *physical* Human-Robot Interaction (pHRI). It is worth noting that cases in which the physical component is completely absent are relatively rare: even with careful trajectory planning and contact-avoidance algorithms, uncertainty and unforeseen human motion create a residual possibility of contact. Examples of pHRI span multiple domains, including industrial tasks like palletization, co-assembly, and load handling; service and personal care tasks such as delivery, mobility support, and patient transfer; and medical applications including guidance, rehabilitation, physiotherapy, and exoskeleton use.

A primary concern in scenarios involving pHRI is ensuring human safety, even under dynamic and partially unknown environmental conditions. While *safety* is often treated as a fundamental property, it is an emergent characteristic of the interaction context: it depends not only on the robot’s behavior but also on the environment, the task, and human actions. For instance, should safety be operationally defined as the prevention of physical injury, or more broadly as the avoidance of pain and discomfort? Furthermore, can a system legitimately be classified as safe if there exists a small but nonzero probability of harm? In some cases, defining or assessing safety becomes a philosophical problem. For example, what can a robot do to remain “safe” if a human suddenly runs toward it and collides, even when the robot itself is stationary and behaving correctly?

To provide practical guidance, the International Organization for Standardization (ISO) bridges the gap between the abstract concept of safety and its operational implementation, adopting a functional perspective in which safety is defined as the ability of a robotic system to operate within limits that reduce risks to humans to an acceptable level. Quantitative limits for mitigating human injury are specified in ISO/TS 15066 (International Organization for Standardization, 2016), which supports human-robot coexistence and explicitly addresses physical interaction through pre-collision guidelines for safe pHRI. While ISO/TS 15066 plays a central role in defining pHRI safety, it contains inherent ambiguities that may lead to inconsistent risk assessments or hazard underestimation. Effective application, therefore, requires careful interpretation, as its assumptions influence both safety evaluation and robot performance. A clear understanding is essential not only to ensure coherent implementation and risk minimization, but also to optimize task execution in real-world scenarios. The work in (Zanella et al., 2026) provides a rigorous analysis of ISO/TS 15066 safety constraints, clarifying their derivation and underlying assumptions, enabling consistent interpretation, addressing underspecified situations, and supporting systematic evaluation of their impact on both safety and performance, ultimately guiding best practices and informed improvements.

Building on the broader critical review in (Zanella et al., 2026), this Perspective Article isolates one central thread: the role of mechanical energy as a unifying quantity for interpreting safety constraints in pHRI. The aim is not to provide a comprehensive review of pHRI safety, nor to introduce new experimental data, but to present the authors’ viewpoint on how energy clarifies several assumptions and ambiguities in ISO/TS 15066. In (Zanella et al., 2026), the role of energy emerged from the broader analysis of safety constraints, but it was not the exclusive focus. Here, we make it the organizing perspective of the manuscript. Specifically, we discuss the ISO/TS 15066 transient-contact constraint in terms of energy conversion up to maximum tissue deformation, clarify the role of impact restitution, examine how energy transfer changes when the contacted body segment can or cannot recoil, and discuss why energy-based metrics are useful for safety assessment and control but insufficient as standalone descriptors of pain perception or sustained-contact risk.

We conclude with a discussion of open challenges and future research directions for leveraging energy-centered metrics to ensure safe and effective human-robot interaction.

## 2 An energetic perspective on safe pHRI

In ISO/TS 15066, the operative limits to be enforced in robot control are derived from the quantitative characterization of human pain. Maximum allowable force and pressure values, measured within average pain tolerance for different anatomical regions, are translated into limits on the mechanical energy that a robot may transfer during contact. The detailed derivation is beyond the scope of this document and is thoroughly presented in (Zanella et al., 2026); however, we briefly outline the procedure here to highlight several relevant considerations. Moreover, the energy-transfer formulation itself is not introduced here as a new safety criterion, but is already part of the informative Annex A of ISO/TS 15066; the present Perspective focuses on its physical interpretation at maximum compression and on its implications for pHRI safety assessment.

Robot dynamics are reduced to an effective mass $m_{R}$ , representing the configuration-dependent reflected inertia at the contact point. The impacted human body region is modeled as an effective mass $m_{H}$ , capturing the inertial properties of the anatomical segment. Tissue deformation at the interface is represented by a linear elastic spring with stiffness k . Consequently, the human-robot interaction is idealized as a collision between two lumped masses connected by a compliant element (see Figure 1). This reduced model follows the simplified transient-contact representation adopted in ISO/TS 15066. It assumes one-dimensional normal impact, a single effective contact point, linear tissue stiffness, and effective masses evaluated along the impact direction, while neglecting friction, shear, contact-area evolution, and nonlinear tissue dynamics. These assumptions, discussed in detail in (Zanella et al., 2026), are retained here only to capture the dominant normal compression mechanism underlying the ISO/TS 15066 transient-contact formulation and to expose its energy balance, thereby supporting the article’s energy-centric perspective. It should be emphasized that the model is not intended to represent nonlinear, viscoelastic, shear, or distributed-contact effects. Within this reduced representation, the effective masses describe the inertia participating along the impact direction, while the linear spring provides a simple force-deformation relation needed to expose the corresponding energy balance. Within this framework, pain-sensitive force thresholds $F_{\max}$ are mapped to a maximum admissible elastic potential energy $U_{s,\max}=\frac{F_{\max}^{2}}{2k}$ that the tissue can safely store during contact-induced deformation, thereby limiting the kinetic energy that the robot (moving at relative pre-collision velocity $v_{0}$ ) can transfer to the human and convert into elastic deformation at impact:

$$\Delta K=\frac{1}{2}\frac{m_{R}m_{H}}{m_{R}+m_{H}}v_{0}^{2}\leq U_{s,\max}.$$ (1)

**FIGURE 1:**
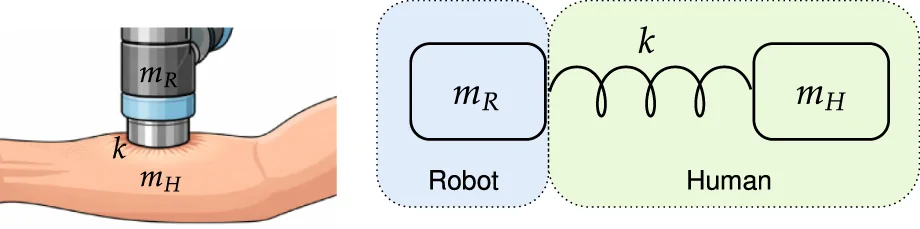
Transient-contact model adopted in ISO/TS 15066, with robot and human effective masses $m_{R}$ and $m_{H}$ . Tissue deformation at the contact point is represented by a linear spring of stiffness k . The model assumes one-dimensional normal impact at a single effective contact point and neglects friction, shear, contact-area evolution, and nonlinear tissue effects.

Here, $\Delta K=K_{0}-K_{*}$ represents the difference between the robot’s initial kinetic energy $K_{0}$ and the kinetic energy of the combined human-robot system after impact $K_{*}$ . This constraint can also be expressed as an explicit limit on the robot’s initial kinetic energy:

$$K_{0,\max}=\frac{m_{R}+m_{H}}{m_{H}}U_{s,\max}.$$ (2)

When the pHRI is modeled in this way, the critical instant for safety evaluation coincides with the moment of *maximum deformation*, at which the biomechanical loading attains its peak value. In this setting, the safety condition is therefore whether the admissible deformation energy is exceeded at maximum compression. The subsequent response, whether elastic restitution or inelastic dissipation, occurs after this peak-load condition has already been reached and therefore does not change the maximum deformation used for the safety assessment. This differs from common literature interpretations that relate pHRI safety to the energy dissipated under a potential inelastic-impact assumption (Rossi et al., 2015; Meguenani et al., 2015): in the present view, whether the subsequent response dissipates energy or returns it is secondary, since it does not affect the maximum deformation already reached. This interpretation relies on the simplifying assumptions of the lumped mass-spring model, which excludes strong damping, viscoelasticity, repeated impacts, secondary collisions, and other effects, thereby highlighting a key limitation of ISO-oriented pHRI safety assessment.

Takeaway 1: The robot must enter the collision with sufficiently low kinetic energy to ensure tissue compression remains within safe limits, independently of whether the impact is elastic or inelastic.

The effective masses of the robot $m_{R}$ and the human $m_{H}$ play a central role in safety assessment, as their relationship determines how impact energy is partitioned. The dependence enters through the reduced mass $\mu =\frac{m_{R}m_{H}}{m_{R}+m_{H}}$ in (1), which governs the kinetic energy available for conversion into elastic deformation. When the robot mass dominates $\left(m_{R}\gg m_{H}\right)$ , only a fraction of the initial robot kinetic energy is stored in the tissue, while the residual energy remains in the robot as post-impact motion. In this case, the admissible initial robot kinetic energy can also be significantly larger than the tissue energy capacity $U_{s,\max}$ . Conversely, when the human effective mass dominates $\left(m_{H}\gg m_{R}\right)$ , nearly all transferred kinetic energy is converted into elastic deformation; indeed, from Equation 1, 2, the admissible initial robot kinetic energy approaches the tissue energy capacity, i.e., $\Delta K\approx K_{0,\max}\approx U_{s,\max}$ . In the limiting case $m_{H}\to \infty$ , the interaction reduces to a *clamped condition*, in which the contacted human body segment is treated as effectively immobilized and cannot recoil. This represents the most conservative scenario: when recoil is possible ( $m_{H}$ finite), only part of the robot’s initial kinetic energy is converted into tissue deformation, whereas preventing recoil makes the tissue energy capacity itself the limiting quantity, so that the safe bound reduces to $K_{0,\max}=U_{s,\max}$ . More specifically, when recoil is possible, part of the pre-impact kinetic energy remains as motion of the coupled human-robot system after maximum compression, so only the relative kinetic-energy component is available for tissue deformation. When recoil is prevented, this mechanism for reducing deformation energy disappears, and the robot kinetic energy itself becomes the conservative quantity to be bounded. Energetically, this distinction is important because ISO/TS 15066 distinguishes quasi-static and transient contacts through empirical scaling of admissible force limits, but does not explicitly reformulate the energy balance for true clamp-like conditions, where the contacted segment cannot recoil. In such cases, the finite- $m_{H}$ interpretation becomes physically inconsistent, and the conservative bound $K_{0,\max}=U_{s,\max}$ is the physically grounded limit, although it may reduce performance as shown numerically in (Zanella et al., 2026). Less conservative bounds are justified only when the interaction can be reliably treated as transient contact with finite human effective mass and possible recoil.

Takeaway 2: Under the potential-clamp assumption, limiting the robot’s kinetic energy to the tissue’s maximum elastic potential energy provides an appropriate constraint to prevent peak forces from being exceeded.

Mechanical energy emerges as a central quantity for translating safety requirements into operational constraints on robot motion, supporting both safety assessment and controller implementation. This raises a key question: *Can focusing exclusively on mechanical energy ensure human safety, bypassing force- or pressure-based measures associated with pain sensitivity?* From a modeling perspective, energy-based thresholds combine the effects of force magnitude and interaction duration into a single scalar quantity, capturing cumulative mechanical load in quasi-static contact. Hence, if admissible energy thresholds could be measured directly, they would reduce the dependence on detailed contact models, tissue-specific stiffness assumptions, and force-to-energy mappings, while providing a global “energy budget” for dynamic safety control that is less tied to specific contact dynamics. However, the usefulness of energy-based metrics depends on the safety objective considered. Energy-based metrics, are useful for injury-oriented assessment, where absorbed mechanical work is related to tissue damage, deformation, and structural failure, and for control-oriented safety constraints, where kinetic energy provides an enforceable pre-contact safety budget. They are insufficient, however, for pain-oriented assessment, which corresponds to the perceptual response to mechanical stimulation and depends on pressure distribution, contact area, strain rate, deformation gradient, and receptor density, and for sustained-contact scenarios, where power, exposure time, loading history, fatigue, and recovery also become relevant. The question above has been explored in the literature (Zanella et al., 2026) by characterizing human safety hazards in terms of the maximum mechanical energy the body can absorb before irreversible damage, shifting attention from instantaneous peak forces to cumulative energy exposure. Consistently with this classification, directly measurable energy-based thresholds (e.g., energy-to-failure) are more naturally related to injury risk than to pain perception, since pain is also affected by local pressure distribution, contact area, strain rate, deformation gradient, and receptor distribution. For example, the same nominal amount of mechanical energy may produce very different pain responses depending on whether the contact is highly localized or distributed over a compliant surface. Furthermore, although injury thresholds exhibit lower inter-subject variability, being derived from structural failure tests or objective criteria, they are less informative for mild or pre-injury conditions, as most data come from post-mortem specimens, anthropomorphic test devices, or accident reconstructions. Pain thresholds, being lower than mild injury thresholds, can thus serve as a conservative safety criterion under the assumption that pressures below the pain threshold will not produce tissue injury.

Takeaway 3: Directly measurable energy-based hazard thresholds capture cumulative mechanical load and provide a global safety “budget,” but correlate with injury rather than pain and are limited for mild conditions.

## 3 Discussion and conclusion

At the core of the safety constraints discussed above lies a simple energetic requirement: the robot must not carry more kinetic energy than can be safely converted into biomechanical loading during contact. As shown in the derivation detailed in (Zanella et al., 2026), pain-sensitive force thresholds can be mapped to a maximum admissible elastic energy stored in the contacted tissue, while robot velocity and effective mass determine the energy available for such deformation. Safe interaction therefore requires bounding the robot’s pre-contact kinetic energy so that the admissible tissue-deformation energy is not exceeded.

In the previous section, we outlined three key observations from this perspective. First, the safety-relevant instant is the moment of maximum deformation, where the relative velocity vanishes and the peak load is reached; the subsequent elastic or inelastic response is therefore secondary. Second, when the contacted body segment can recoil, only part of the robot’s kinetic energy is converted into tissue deformation, whereas if recoil is prevented, nearly all of it may be stored in the tissue, making $K_{0,\max}=U_{s,\max}$ the appropriate conservative bound. Third, energy-based thresholds capture cumulative mechanical load, but safe energy limits for pain or mild pre-injury contacts cannot be measured directly and must instead be derived from force and pressure limits.

Future work should build on this energetic interpretation of safety in pHRI while addressing its current limitations. A first direction is the development of biomechanics-aware human–robot interaction models that go beyond the lumped mass–spring approximations underlying ISO/TS 15066. Such models should combine musculoskeletal dynamics, data-driven soft-tissue models, and reduced-order representations such as strain maps to capture posture-dependent dynamics, distributed compliance, multi-contact interactions, and tissue-specific effects, while estimating internal safety variables such as tissue deformation, muscle strain, excessive joint loading, and fatigue in real time. A natural modeling direction for such energy-aware safety assessment is to use frameworks that make energy storage, dissipation, and exchange explicit, rather than treating energy only as a scalar bound on robot motion. Port-Hamiltonian formulations are particularly suited to this objective because they describe physical systems in terms of energy storage, dissipation, and power exchange through well-defined interaction ports (Duindam et al., 2009). In pHRI, this structure makes it possible to represent the robot, the human body, and the environment as interconnected energetic subsystems, exposing where mechanical energy is injected, stored, dissipated, or transferred during contact (Geravand et al., 2016). This is especially relevant for safety, since it provides a principled way to track energy flows along the robot kinematic chain and into human body segments, rather than evaluating contact only through local force or velocity limits. Compared with energy-based control methods that impose global kinetic-energy or velocity bounds, the port-Hamiltonian structure can localize energy exchange at specific interaction ports and therefore provides a more direct route to enforceable constraints on the energy and power transmitted to the human. Moreover, the modularity of port-Hamiltonian models supports the interconnection of heterogeneous components, such as rigid robot dynamics, compliant tissues, musculoskeletal joints, and external constraints, while preserving a physically meaningful energy balance. This could enable safety controllers to impose constraints directly on exchanged energy and power at the human-robot ports, linking control-oriented guarantees such as passivity to biomechanical safety metrics. From a control perspective, future methods should move beyond scalar global energy limits toward context-aware safety constraints that jointly regulate energy and power flow. In pre-collision scenarios, this requires adapting kinetic-energy bounds to the robot configuration, the possible contact location, the impact direction, and the admissible loading of the exposed body region. During contact, while energy bounds limit the total mechanical load exchanged, power constraints capture how rapidly this energy is delivered and are therefore critical for impulsive or sustained human-robot interaction. Port-Hamiltonian formulations can support this transition by providing a structured decomposition of energy flows, making it possible to identify the components of motion that are relevant for a given interaction without unnecessarily constraining the remaining dynamics. Finally, safety assessment should also be extended to pHRI scenarios in which contact is intentional and maintained over time, rather than accidental and transient. The energy constraints discussed in the main part of this article apply primarily to isolated transient impacts, where the dominant safety-relevant event is maximum deformation or peak contact load. Sustained-contact interactions, such as rehabilitation, exoskeletons, personal assistance, service robotics, or collaborative load handling, are different: they cannot be assessed only through a single pre-impact kinetic-energy bound. In such cases, risk depends on the continuous exchange of energy and power, as well as on cumulative biomechanical effects. For contacts involving relative motion, mechanical power provides a natural metric for quantifying continuous energy exchange; for contacts with little or no relative motion, such as clamping, support, or intentional stops, safety assessment should also consider strain accumulation, stored elastic energy over time, exposure duration, loading history, fatigue, and possible tissue recovery. These additional metrics would provide a basis for future enforceable safety conditions in scenarios not adequately covered by current collision-oriented or static force-based methodologies.

## Data Availability

The original contributions presented in the study are included in the article/supplementary material, further inquiries can be directed to the corresponding author.
